# Does femoral stem choice associate with survivorship and clinical outcomes after conversion total hip arthroplasty? A retrospective analysis and novel treatment algorithm

**DOI:** 10.1007/s00402-025-06181-4

**Published:** 2026-02-24

**Authors:** Troy D. Bornes, Daniel Alexander Driscoll, Christopher G. Anderson, Delano R. Trenchfield, Mohammed El-Hassan, Ittai Shichman, Elizabeth B. Gausden, Allina A. Nocon, Peter K. Sculco

**Affiliations:** 1 Complex Joint Reconstruction Center, Adult Reconstruction and Joint Replacement Service, Hospital for Special Surgery, New York, USA; 2 Southern California Orthopedic Institute, Van Nuys, USA

**Keywords:** Conversion total hip arthroplasty, Proximal femoral fracture, Cephalomedullary nail, Sliding hip screw

## Abstract

**Background:**

Conversion total hip arthroplasty (cTHA) after prior proximal femoral fracture fixation can be performed using different femoral stems. This study aimed to (1) determine if initial fracture fixation or pattern associated with stem design in cTHA, (2) evaluate whether stem type was associated with survivorship and outcomes, and (3) propose a radiographic measurement to guide femoral stem selection.

**Methods:**

We retrospectively reviewed 51 patients who underwent cTHA from 2016 to 2020, including 44 patients with previous cephalomedullary nail (CMN; 86.3%) and 9 patients with previous sliding hip screw (SHS; 13.7%). Stems included 32 uncemented diaphyseal-engaging (62.7%), 10 uncemented metaphyseal-engaging (19.6%), and 9 cemented (17.6%). Outcomes assessed were fracture pattern, fixation construct, stem type, survivorship, post-operative complications, and patient-reported outcome measures. Preoperative radiographs were evaluated for diaphyseal width (D), distance from greater trochanter to most distal pre-isthmic screw (GS), and ratio of GS/D (GSD). These measures were used to construct an algorithm for stem selection at the time of cTHA. Mean follow-up was 69 months. Survivorship free from stem-related and all-cause failure was calculated using Kaplan-Meier analysis.

**Results:**

Diaphyseal-engaging uncemented stems were used more often after CMN fixation, while metaphyseal-engaging uncemented stems predominated after SHS fixation. Diaphyseal-engaging stems predominated in previous intertrochanteric, subtrochanteric, or complex fractures, while metaphyseal-engaging uncemented stems were more common in femoral neck fractures. Survivorship free from stem-related failure was 100% and from all-cause reoperation was 89%. Patients who received cemented stems were older, more likely to require transfusion or prolonged use of an assistive walking device. No significant differences were observed in complications, clinical outcomes or PROMs between stem groups. Higher GSD ratios correlated with diaphyseal-engaging stem use in SHS and long-CMN cases.

**Conclusion:**

Femoral stem fixation type in cTHA correlated with prior fracture pattern and fixation construct. Survivorship free from stem-related failure requiring stem revision was 100% and survivorship free from all-cause failure was 89%. We propose a novel algorithm that may assist surgeons in selecting femoral stem at the time of cTHA using different patient-specific and radiographic factors.

**Supplementary Information:**

The online version contains supplementary material available at 10.1007/s00402-025-06181-4.

## Introduction

Proximal femur fractures, including subcapital, intertrochanteric and subtrochanteric fracture types, are common injuries with an annual incidence that is likely to increase secondary to an aging population [1, 2]. Depending on fracture pattern and surgeon preference, surgical treatment may include a sliding hip screw (SHS), a short cephalomedullary nail (CMN) or a long CMN [3–6]. A subgroup of patients with these surgically treated fractures go on to develop osteoarthritis, avascular necrosis, nonunion, or malunion and require conversion to a total hip arthroplasty (cTHA) [7]. 

Survivorship following cTHA has been shown to be lower than primary THA (pTHA) [8–14]. Relatively high rates of complications have been reported after cTHA compared to pTHA and suggest that cTHA is more comparable to revision THA than pTHA [15–17]. It has also previously been demonstrated that patients who undergo cTHA who had fracture fixation with CMN have worse functional outcomes compared to patients who receive extramedullary fixation, likely due to the involvement of the abductors [8, 15, 18, 19]. 

Although there are fairly robust data in regard to the impact of prior fracture fixation on outcomes, there is a relative paucity of literature evaluating the impact of femoral stem type selection at the time of cTHA on survivorship and patient-reported outcome measures (PROMs) [20]. Multiple different stem types—including metaphyseal-engaging stems, diaphyseal-engaging stems, and cemented stems—have been used in cTHA [19, 21–24]. It is currently unclear whether stem type affects survivorship, patient-reported outcome measures (PROMs), or functional outcomes after cTHA. In addition to a lack of evidence regarding the impact of femoral stem type in cTHA on survivorship and PROMs, there are also limited data on whether the proximal femoral fracture pattern or fracture fixation construct impacts femoral stem selection at the time of cTHA. Finally, there is limited published guidance for surgeons on stem selection at the time of cTHA.

This study aimed to: (1) characterize femoral stem types used in cTHA after proximal femoral fracture fixation and assess associations with prior fracture patterns and fixation methods; 2) evaluate whether cTHA stem type associated with outcomes or survivorship, and 3) propose a novel preoperative radiographic measurement to guide stem choice in cTHA.

## Methods

Ethical approval for this retrospective study was obtained from an institutional review board. Within an institutional arthroplasty registry of a high-volume arthroplasty service, patients were identified using procedure code and name. The following inclusion criteria were used: (1) cTHA performed from January 1, 2016 to December 31, 2020; (2) previous diagnosis of a proximal femoral fracture classified by the Arbeitsgemeinschaft für Osteosynthesefragen (AO)/Orthopaedic Trauma Association (OTA) system in the femoral neck (31-B), trochanteric region (31-A), and subtrochanteric region of the femoral shaft (32 A-C, proximal third) within 5 cm of the lesser trochanter treated with closed or open reduction and internal fixation using a cephalomedullary nail (CMN) or sliding hip screw (SHS) [25]. Exclusion criteria were: (1) previous diagnosis of femoral head fracture (AO/OTA 31-C), femoral shaft fracture (AO/OTA 32 A-C) distal to the subtrochanteric region, or distal femoral fracture (AO/OTA 33 A-C); (2) neoplasm-associated fracture; or (3) previous non-operative treatment of fracture. Operative reports, physician notes, and imaging were reviewed to confirm that patients met inclusion criteria.

### Data collection and variables of interest

Chart review was performed to obtain demographics and surgical variables including date of cTHA, primary diagnosis, implants, operative time, and postoperative complications. Duration from surgery to last follow-up, need for revision, and reason for revision were evaluated. In addition, charts were reviewed at last follow up to ascertain the following clinical variables: presence of anterior thigh pain, use of ambulatory assistive device, presence of limp, presence of clinical limb length discrepancy, postoperative wound complication, range of motion (ROM). Preoperative and 1-year postoperative Hip dysfunction and Osteoarthritis Outcome Score for Joint Replacement (HOOS, JR.) were prospectively collected.

### Patients

There were 51 patients identified after applying our inclusion and exclusion criteria including 19 male patients (37.3%) and 32 female patients (62.7%) (Table 1). Mean age was 73 years and mean body mass index was 27.5. All cTHAs were performed using a posterior approach to the hip. Stems used at the time of cTHA included 32 uncemented diaphyseal-engaging stems (62.7%), 10 uncemented metaphyseal-engaging stems (19.6%), and 9 cemented stems (17.6%). There was a significant difference between stem groups with respect to age, with cemented stems used in older patients. Liners used included 25 dual mobility liners (49.0%), 25 standard polyethylene liners (49.0%), and 1 constrained liner (2.0%). Mean time to follow-up was 69 months (range, 46 to 102).

**Table 1:** Patient demographics based on femoral stem implant used at time of conversion total hip arthroplasty

Variables	All (n = 51)	Diaphyseal-engaging stems (n = 32)	Metaphyseal-engaging stems (n = 10)	Cemented stems (n = 9)	P value
Age (Years), mean (range)	73.4 (40–92)	73.6 (42–86)	64.6 (40–92)	82.2 (60–90)	**0.005**
Men, *n* (%)	19 (37.3%)	11 (34.4%)	5 (50.0%)	3 (33.3%)	0.71
BMI (kg/m^2^), mean (range)	27.5 (16.1–41.6)	27.3 (16.8–39.4)	28.9 (16.1–41.6)	26.9 (17.8–35.9)	0.29
Diabetes, *n* (%)	43 (84.3%)	28 (87.5%)	7 (70.0%)	8 (88.9%)	0.47
Smoking status, n (%)					
Current	3 (5.9%)	2 (6.3%)	1 (10.0%)	0 (0.00%)	
Former	25 (49.0%)	18 (56.3%)	5 (50.0%)	2 (22.2%)	0.26
Never	23 (45.1%)	12 (37.4%)	4 (40.0%)	7 (77.8%)	
ASA score, *n* (%)					
II	29 (56.9%)	19 (59.4%)	7 (70.0%)	3 (33.3%)	0.29
III	22 (43.1%)	13 (40.6%)	3 (30.0%)	6 (66.7%)	
Time to cTHA (months), mean (range)	39.8 (0.9–194)	44.7 (0.9–194)	35.6 (10.9 −188.6)	25.4 (7.7–78.1)	0.93
Follow-up from cTHA (months), mean (range)	69.2 (46.1–101.6.1.6)	65.8 (46.1–99.7)	69.5 (48.3–93.7)	80.9 (59.5–101.6.5.6)	0.09
Operative time, minutes (SD)	181.0 (63.0)	186.4 (64.8)	157.4 (67.2)	188.1 (50.6)	0.4

*cTHA* Conversion total hip arthroplasty, *ASA* American society of anesthesiologists, *THA* Total hip arthroplasty, *BMI* Body mass index, *SD* Standard deviation

P values calculated using analysis of variance, Chi-square or Fisher-Freeman-Halton to compare groups

Significant P values in bold

### Prior fixation and indication for conversion THA

Proximal femoral fracture pattern and fixation construct used in previous fracture surgery are shown in Table 2. Fracture fixation constructs included 44 CMNs (86.3%) and 7 SHSs (13.7%). Primary diagnoses leading to cTHA are outlined in Supplemental Table 1. Radiographic signs of osteoarthritis were noted before cTHA in 46 cases, regardless of the primary diagnosis leading to conversion THA (90%; Supplemental Table 2). In the 5 patients without radiographic signs of osteoarthritis, there were 4 patients with primary diagnosis of nonunion and one patient with primary diagnosis of malunion.

**Table 2:** Fixation construct used in previous proximal femoral fracture surgery based on fracture pattern

Fracture fixation construct used in previous fracture surgery	Number of patients (%), subgroups based on fracture pattern at fracture surgery					
	All patients (*n* = 51)	Neck fracture (*n* = 13)	IT fracture (*n* = 32)	ST fracture (*n* = 2)	Complex/multiple (*n* = 4)	*P* value
Long cephalomedullary nail	17 (33.3%)	3 (23.1%)	9 (28.1%)	2 (100.0%)	3 (75.0%)	**< 0.001**
Short cephalomedullary nail	27 (52.9%)	3 (23.1%)	23 (71.9%)	0 (0.0%)	1 (25.0%)	
Sliding hip screw	7 (13.7%)	7 (53.8%)	0 (0.0%)	0 (0.0%)	0 (0.0%)	

*IT* Intertrochanteric, *ST* Subtrochanteric, *THA* Total hip arthroplasty

P values calculated using Fisher-Freeman-Halton to compare groups

Significant P values in bold

### Radiographic evaluation of fracture pattern, loosening, and stem subsidence

A radiographic review of preoperative radiographs was performed by a fellowship-trained surgeon to evaluate proximal femoral fracture pattern (femoral neck fracture, intertrochanteric fracture, subtrochanteric fracture, and complex/multiple proximal femoral fracture), fracture fixation implant, and pre-operative diagnoses. Femoral stem fixation and loosening on radiography were evaluated by two surgeons with fellowship training in arthroplasty at final follow-up compared with initial postoperative radiographs [26–29]. Stem subsidence was evaluated on calibrated radiographs by measuring the difference in distance between the greater trochanter and femoral stem (shoulder) at final follow-up compared to initial postoperative radiographs [30]. Subsidence ≥5 mm was deemed to be significant subsidence.

### Radiographic evaluation of stem bypass of prior screw holes

**Fig. 1:**
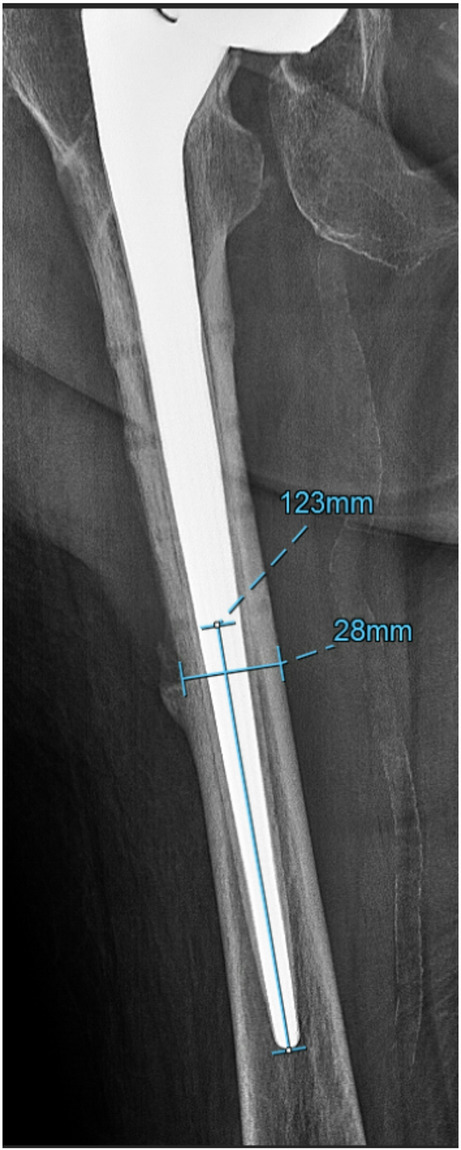
Representative radiographs showing measurement of SSD (stem to screw distance divided by diaphyseal width) measurement. Vertical measurement represents the distance of femoral stem that bypasses the most distal screw hole proximal to the femoral isthmus (SS). Horizontal measurement represents diaphyseal width

Postoperative radiographs were evaluated by a fellowship-trained orthopedic surgeon to calculate the distance of femoral stem that bypassed the most distal screw hole proximal to the femoral isthmus (Fig. 1; SS [*S*tem to *S*crew distance]). The SS (in mm) was then divided by the diaphyseal width of the femur (in mm) to create a ratio, defined as “SSD” (SS divided by *D*iaphyseal width).

### Novel radiographic measurements and algorithm creation

**Fig. 2:**
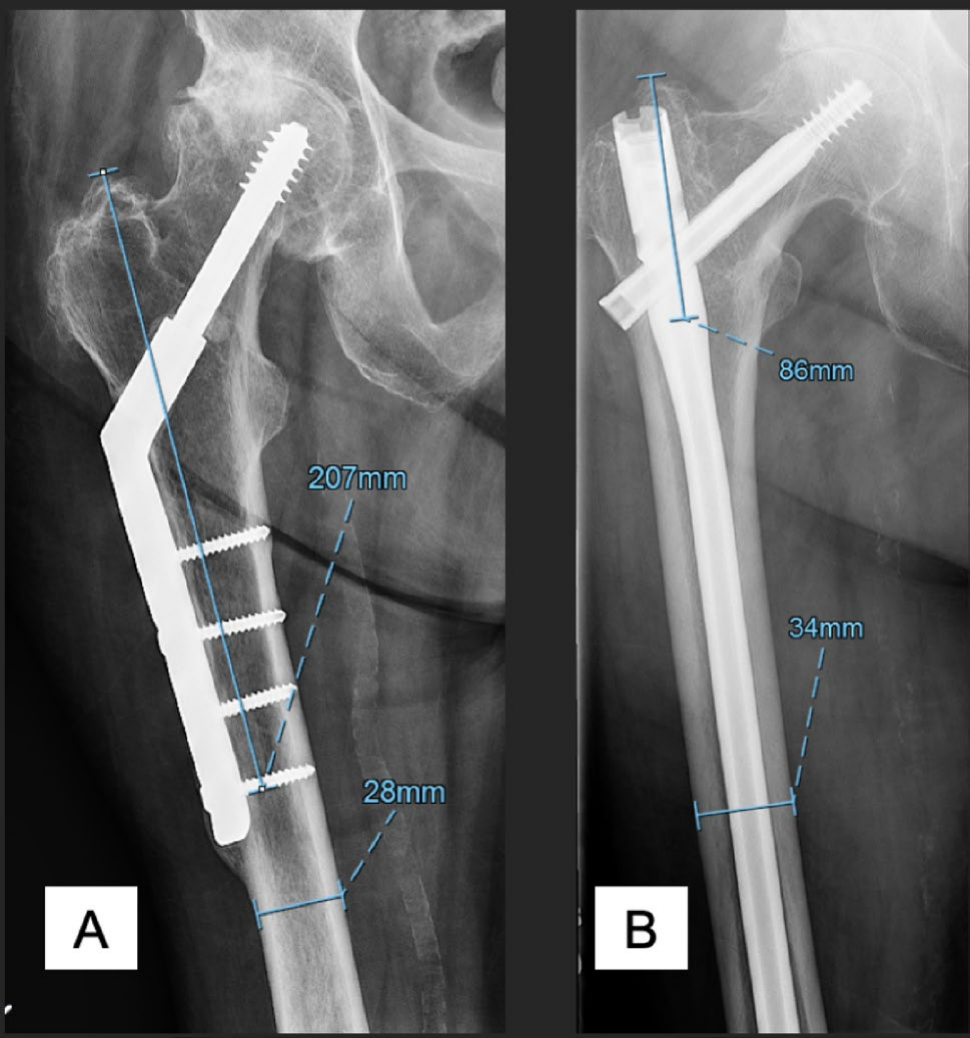
Representative radiographs showing measurement of GSD (distance from the proximal aspect of the greater trochanter to the most distal screw proximal to the femoral isthmus divided by the diaphyseal width) in patients with sliding hip screw and long cephalomedullary nail constructs. GS represents distance from greater trochanter to most distal screw proximal to the femoral isthmus (vertical measurement). Horizontal measurement represents diaphyseal width **A**. Measurement of GSD in a patient with sliding hip screw fixation **B**. Measurement of GSD in a patient with long cephalomedullary nail fixation

Preoperative radiographs were evaluated by a fellowship-trained orthopedic surgeon to calculate a novel measurement, the distance from the proximal aspect of the greater trochanter to the most distal screw proximal to the femoral isthmus (Fig. 2; defined as “GS” [*G*reater trochanter to *S*crew distance]). The diaphyseal width was measured at the femoral isthmus. The GS was then divided by the diaphyseal width of the femur to create a ratio, GSD. This ratio-based approach was intentionally used to minimize the effects of radiographic magnification, as dividing by the diaphyseal width mitigate any potential variation in image scaling. GSD was then compared among patients to assess whether there was an association with stem choice. A novel treatment algorithm to guide femoral stem selection was then constructed using this measurement along with other patient-specific data.

### Data analyses

Descriptive data were reported as mean values with standard deviations. All-cause failure was defined as the need for reoperation for any cause, while femoral stem-related failure was defined as the need for femoral stem revision for aseptic loosening or implant fracture. Survivorship was calculated using the Kaplan-Meier method with 95% confidence interval (CI) reported. Log-rank test was used to identify differences between curves. Chi-squared tests or Fischer-Exact tests were used to compare categorical variables. Mann-Whitney U or t-tests were used to compare continuous variables. *P* values < 0.05 were considered statistically significant. Statistical analyses were performed using SAS version 9.3 (SAS Institute Inc., Cary, NC).

## Results

### Femoral stem fixation used in conversion THA

Femoral stem fixation used in cTHA was significantly different in patients treated with different fixation constructs during fracture surgery (Table 3; *P* = 0.01). Diaphyseal-engaging uncemented stems were the predominant stem type used in patients with previous CMN (30/44, 68%) and were used at a higher rate in this group than in those with SHS fixation (2/7, 29%; *P* = 0.04). In contrast, metaphyseal-engaging uncemented stems were the predominant type used after SHS (4/7, 57%) and were used at a higher rate in this group than in those with CMN fixation (6/44, 14%; *P* = 0.007). Cemented stems appeared to be used at a higher frequency in patients with previous long CMN fixation (5/17, 29%) compared to short CMN and SHS fixation (4/34, 12%) although a significant difference was not demonstrated (*P* = 0.1).

**Table 3:** Femoral stem implanted in conversion total hip arthroplasty based on fixation construct used in previous proximal femoral fracture surgery

Femoral stem type used in conversion THA	Number of patients (%), subgroups based on fracture fixation construct used in previous fracture surgery					
	All patients (*n* = 51)	All CMN (*n* = 44)	Long CMN (*n* = 17)	Short CMN (*n* = 27)	SHS (*n* = 7)	*P* value
*Uncemented*	42 (82.3%)	36 (81.8%)	12 (70.6%)	24 (88.8%)	6 (85.7%)	**0.01**
Diaphyseal-engaging	32 (62.7%)	30 (68.2%)	8 (47.1%)	22 (81.5%)	2 (28.6%)	
Modular	17 (33.3%)	16 (36.4%)	5 (29.4%)	11 (40.7%)	1 (14.3%)	
Monobloc	15 (29.4%)	14 (31.8%)	3 (17.6%)	11 (40.7%)	1 (14.3%)	
Metaphyseal-engaging	10 (19.6%)	6 (13.6%)	4 (23.5%)	2 (7.4%)	4 (57.1%)	
Single-taper/blade	4 (7.8%)	3 (6.6%)	2 (11.8%)	1 (3.7%)	1 (14.3%)	
Double-taper/fit and fill	6 (11.8%)	3 (6.8%)	2 (11.8%)	1 (3.7%)	3 (42.9%)	
*Cemented*	9 (17.6%)	8 (18.2%)	5 (29.4%)	3 (11.1%)	1 (14.3%)	

*CMN* Cephalomedullary nail, *SHS* Sliding hip screw, *THA* Total hip arthroplasty

P value calculated using Fisher-Freeman-Halton to compare diaphyseal-engaging stem vs. metaphyseal-engaging stem vs. cemented stem groups

Significant P values in bold

The type of femoral stem fixation used in cTHA was significantly different in patients with different initial proximal femoral fracture patterns (Table 4; *P* = 0.008). Diaphyseal-engaging uncemented stems were the type used in patients with previous intertrochanteric, subtrochanteric, or multiple/complex fractures (28/38, 74%) and were used at a higher rate in this group than in those with femoral neck fractures (4/13, 31%; *P* < 0.001). Metaphyseal-engaging uncemented stems were the predominant type used in patients with previous femoral neck fractures (7/13, 54%) and were used at a higher rate in this group than in those with other fractures (3/38, 8%; *P* < 0.001).

**Table 4:** Femoral stem implanted in conversion total hip arthroplasty based on fracture pattern at previous proximal femoral fracture surgery

Femoral stem type used in conversion THA	Number of patients (%), subgroups based on fracture pattern at fracture surgery						
	All patients (*n* = 51)	Neck fracture (*n* = 13)	IT fracture (*n* = 32)	ST fracture (*n* = 2)	Complex/multiple (*n* = 4)	*P* value	
*Uncemented*	42 (82.3%)	11 (84.6%)	26 (81.3%)	2 (100.0%)	3 (75.0%)	**0.008**	
Diaphyseal-engaging	32 (62.7%)	4 (30.8%)	25 (78.1%)	1 (50.0%)	2 (50.0%)		
Modular	17 (33.3%)	1 (7.7%)	14 (43.8%)	1 (50.0%)	1 (25.0%)		
Monobloc	15 (29.4%)	3 (23.1%)	11 (34.4%)	0 (0.0%)	1 (25.0%)		
Metaphyseal-engaging	10 (19.6%)	7 (53.8%)	1 (3.1%)	1 (50.0%)	1 (25.0%)		
Single-taper/blade	4 (7.8%)	3 (23.1%)	0 (0.0%)	1 (50.0%)	0 (0.0%)		
Double-taper/fit and fill	6 (11.8%)	4 (30.8%)	1 (3.1%)	0 (0.0%)	1 (25.0%)		
*Cemented*	9 (17.6%)	2 (15.4%)	6 (18.8%)	0 (0.0%)	1 (25.0%)		

*IT* Intertrochanteric; *ST* Subtrochanteric; *THA* Total hip arthroplasty

P value calculated using Fisher-Freeman-Halton Test to compare diaphyseal-engaging vs. metaphyseal-engaging vs. cemented

Significant P values in bold

### Survivorship

Survivorship free from stem-related failure requiring stem revision was 100% calculated using the Kaplan-Meier method with a mean follow-up of 69 months (Supplemental Table 4). With respect to all-cause failure requiring reoperation, 5 failures occurred within the first 100 days after cTHA (3 infection and 2 for instability; Supplemental Table 3). One patient treated for infection went on to sustain a Vancouver C periprosthetic fracture that was treated with open reduction and internal fixation with a lateral femoral plate. Survivorship free from all-cause failure was 89% (CI 79–98%; Supplemental Fig. 1). There were no significant differences in survivorship noted between uncemented and cemented stems (*P* = 0.8; Supplemental Fig. 2), diaphyseal-engaging and metaphyseal-engaging uncemented stems (*P* = 1.0; Supplemental Fig. 3) and modular diaphyseal-engaging and monobloc diaphyseal-engaging stems (*P* = 0.7; Supplemental Fig. 4).

### Postoperative complications

There were 15 patients (29.4%) who received transfusion of at least one unit of packed red blood cells postoperatively. Rates of transfusion differed between stem groups significantly (*P* = 0.02; Table 5**)**. Rates of transfusion for patients with metaphyseal-engaging stems, diaphyseal-engaging stems, and cemented stems were 10.0%, 25.0% and 66.7%, respectively.

**Table 5:** Postoperative complications and clinical outcomes based on femoral stem implant used at time of conversion total hip arthroplasty

Variables	All (*n* = 51)	Diaphyseal-engaging stems (*n* = 32)	Metaphyseal-engaging stems (*n* = 10)	Cemented stems (*n* = 9)	*P* Value
Transfusion, *n* (%)	15 (29.4%)	8 (25.0%)	1 (10.0%)	6 (66.7%)	**0.02**
Periprosthetic fracture, *n* (%)	1 (2.0%)	1 (3.1%)	0 (0%)	0 (0%)	1.0
Periprosthetic joint infection, *n* (%)	3 (5.9%)	2 (6.3%)	1 (10.0%)	0 (0%)	0.8
Reoperation, *n* (%)	5 (9.8%)	3 (9.4%)	1 (10.0%)	1 (9.1%)	1.0
Stem revision, *n* (%)	0 (0%)	0 (0%)	0 (0%)	0 (0%)	1.0
Wound complication, *n* (%)	4 (7.8%)	3 (9.4%)	1 (10.0%)	0 (0%)	1.0
Anterior thigh pain, *n* (%)	2 (3.9%)	2 (6.25%)	0 (0.00%)	0 (0%)	1.0
Clinical limp, *n* (%)	16 (31.4%)	9 (28.1%)	4 (40.0%)	3 (33.3%)	0.8
Clinical limb length discrepancy, *n* (%)	4 (7.8%)	2 (6.3%)	2 (20.0%)	0 (0%)	0.3
Preoperative assistive device use, *n* (%)	38 (74.5%)	21 (65.6%)	8 (80.0%)	9 (100%)	0.13
Postoperative assistive device use, *n* (%)	27 (52.9%)	12 (37.5%)	6 (60.0%)	9 (100%)	**0.003**
Postoperative Hip Flexion, mean, mean (SD)	97.8 (9.12)	97.9 (9.73)	97.5 (6.55)	98 (10.9)	1.0
Postoperative Hip External Rotation, mean (SD)	34.5 (9.98)	34.3 (8.05)	36.3 (15.9)	33 (9.75)	0.8

Variables	All	Uncemented stems		Cemented stems	P Value
Preoperative HOOS, JR., mean (SD)	49.6 (19.1)	52.2 (15.4)		27.9 (39.5)	0.5
Postoperative HOOS, JR., mean (SD)	79.4 (16.0)	79.5 (16.4)		78.4 (19.2)	0.9
Percent of patients achieving 10.9 increase in HOOS, JR. (MCID)	72.7%	70.0%		100.0%	1.0

NB: Data above for patient reported outcomes represents 20 patients (39.2% of overall study cohort). Subgroup analysis with division between metaphyseal-engaging and diaphyseal-engaging uncemented stems not shown due to small sample size

*SD* Standard deviation, *MCID* Minimum clinically important difference

P values calculated using analysis of variance, Chi-square or Fisher-Freeman-Halton, independent sample T-test, Mann-Whitney U test, or Fisher’s exact test to compare groups

Significant P values in bold

There were 4 patients (7.8%) with a postoperative wound complication, 1 patient (2.0%) who sustained a periprosthetic fracture following a fall due to syncope, 3 patients (5.9%) with periprosthetic joint infection, and 5 patients (9.8%) who underwent at least one reoperation (Table 5, Supplemental Table 4). There were no differences in rates of these complications between femoral stem groups.

### Clinical outcomes

At most recent follow-up of 69 months, there were 2 patients (3.9%) with documented anterior thigh pain, 16 patients with presence of limp (31.3%), and 4 patients (7.8%) with a clinical limb length discrepancy. There were no significant differences in rates of these clinical outcomes regardless of stem choice at the time of cTHA (Table 5).

There were 37 patients (72.5%) who used a walking assistive device prior to cTHA and 27 patients (52.9%) who used a walking assistive device postoperatively (Table 5). Rates of a walking assistive device use were significantly different between stem groups postoperatively (*P* = 0.003) but not preoperatively (*P* = 0.1). There were 6 patients (5 patients with diaphyseal-engaging stems and 1 patient with a metaphyseal-engaging stem) who began use of a walking assistive device postoperatively after not using one prior to cTHA. There were 17 patients (14 with diaphyseal-engaging stems and 3 with metaphyseal-engaging stems) who used a walking assistive device preoperatively and went on to stop use postoperatively. All 9 patients with cemented stems (100%) used an assistive device preoperatively and continued use postoperatively.

Mean postoperative hip flexion was 97.8 degrees and mean postoperative hip external rotation was 34.5 degrees (Table 5). There were no significant differences in hip motion between femoral stem groups.

### Patient-reported outcome measures

At least one HOOS, JR. score was available for 20 patients (39.2%). Mean preoperative HOOS, JR was 49.6 and mean postoperative HOOS, JR was 79.4 (Table 5). Among patients with both preoperative and 1-year postoperative HOOS, JR available, 72.7% were able to achieve the minimum clinically important difference [MCID] of a 10.9 point increase in HOOS, JR in revision THA [31]). There were no differences in preoperative HOOS, JR., postoperative HOOS, JR. or ability to achieve MCID between cemented and uncemented stem groups.

### Radiographic evaluation for stem bypass

There were 35 patients (68.6%) with SSD ≥ 2 and 47 patients (90.4%) with SSD ≥ 1 (Table 6). Among patients with cementless stems, 29 patients (69.0%) had SSD ≥ 2. There was no difference between stem treatment groups regarding number of patients with SSD ≥ 2, SSD ≥ 1, and mean SSD. Mean SSD was 2.3 for patients with long CMN compared to 3.5 in patients with short CMN and 3.5 in patients with SHS; this difference approached, but did not achieve, significance (*P* = 0.08). Of note, the one patient with postoperative periprosthetic fracture had a calculated SSD of 6.5, indicating substantial engagement of stem distal to prior screw fixation.

**Table 6:** Radiographic evaluation of stem bypass of prior screw holes in conversion total hip arthroplasty

	All patients (*n* = 51)	Diaphyseal stem cohort (*n* = 32)	Metaphyseal stem cohort (*n* = 10)	Cemented stem cohort (*n* = 9)	*P* value
Number of patients with SS/D ≥ 2, *n* (%)	35 (68.6%)	22 (68.8%)	7 (70.0%)	6 (66.7%)	1.0
Number of patients with SS/D ≥ 1, *n* (%)	47 (90.4%)	30 (93.8%)	9 (90.0%)	8 (88.9%)	0.6
Mean SSD (range)	3.1 (0.1–7.5)	3.2 (0.1–7.5)	3.1 (0.4–4.8)	2.6 (0.4–6.6)	0.7

	All patients (n = 51)	Long CMN (n = 17)	Short CMN (n = 27)	Sliding hip screw (n = 7)	P value
Number of patients with SS/D ≥ 2, *n* (%)	35 (68.6%)	11 (64.7%)	18 (66.7%)	6 (85.7%)	0.7
Number of patients with SS/D ≥ 1, *n* (%)	47 (90.4%)	16 (94.1%)	25 (92.6%)	6 (85.7%)	0.6
Mean SSD (range)	3.1 (0.1–7.5)	2.3 (0.1–4.4)	3.5 (0.4–7.5)	3.5 (0.4–6.6)	*0.08*

*CMN* Cephalomedullary nail

*SS/D* Stem to screw distance divided by diaphyseal width

P value calculated for Fisher’s exact test or analysis of variance as appropriate

P values that approach significance (< 0.1) are underlined

### Postoperative stem subsidence

In the radiographic evaluation of femoral stem fixation, mean stem subsidence was 1.2 ± 1.8 mm (range 0–10 mm). Subsidence was ≥5 mm in 4 patients and ≥10 mm in 1 patient; all patients with subsidence greater than 5 mm had diaphyseal-engaging uncemented stems. There was no significant difference noted in subsidence between different stem fixation types (Supplemental Table 5). No stems had qualitative signs of loosening on radiographs.

### Novel radiographic measurements and treatment algorithm

Among patients who received a cementless stem and did not have a malunion/nonunion or complex fracture, patients who received diaphyseal stem had a mean GSD of 4.3 and patients who received a metaphyseal stem had a mean GSD of 4.0 (Table 7). Patients with SHS who received diaphyseal-engaging stems had significantly higher GSD than SHS patients who received metaphyseal-engaging stems (2.5 vs. 2.0; *P* = 0.02). Patients with long CMN who received diaphyseal-engaging stems had significantly higher GSD than long CMN patients who received metaphyseal-engaging stems (6.2 vs. 4.8; *P* = 0.04).

**Table 7:** Radiographic determinants of metaphyseal vs. diaphyseal stem selection

Group	Mean GSD ratio		
	Diaphyseal stem cohort	Metaphyseal stem cohort	*P* value
All patients	4.28	3.98	0.3
Short CMN	4.71	5.43	0.2
Long CMN	2.45	1.98	**0.02**
SHS	6.24	4.76	**0.04**

Cemented stem patients, malunion, nonunion, and complex fractures removed

GSD ratio: Distance from proximal aspect of greater trochanter to most distal screw proximal to or at the isthmus divided by the isthmic diameter

*THA* Total hip arthroplasty, *CMN* Cephalomedullary nailing, *SHS* Sliding hip screw

P value calculated for Student’s T-test

Significant P values in bold

**Fig. 3:**
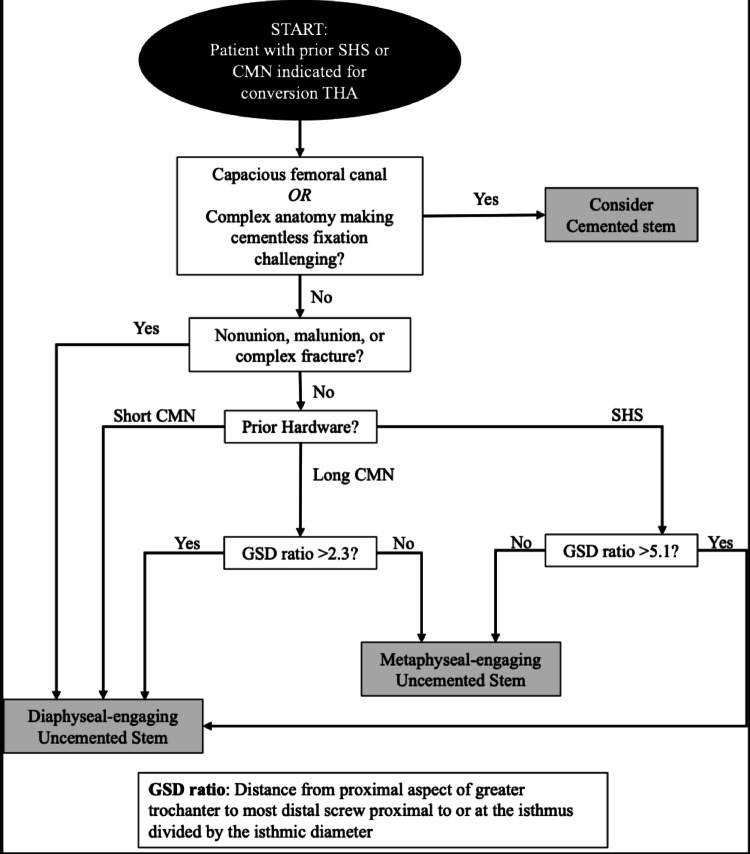
Proposed treatment algorithm for femoral stem choice in conversion total hip arthroplasty in patient with prior sliding hip screw or cephalomedullary nailing

For patients with SHS who were treated with cementless stems, a GSD threshold of 5 correctly predicted stem type in all 6 cases, with values of 5 or greater associated with the use of a diaphyseal-engaging stem and values below 5 associated with a metaphyseal-engaging stem. For long CMN patients who were treated with cementless stems, a GSD threshold of 2.3 had 90% accuracy for predicting stem use (9/10 stems predicted correctly) with GSD of 2.3 or greater predicting diaphyseal-engaging stem and GSD < 2.3 predicting metaphyseal-engaging stem. These preliminary thresholds were incorporated into a prototype treatment algorithm to guide femoral stem selection during conversion THA (Fig. 3).

## Discussion

In this study, we found that femoral stem fixation type was significantly associated with prior fracture fixation construct and fracture pattern, with diaphyseal-engaging stems more commonly used following CMN fixation and metaphyseal-engaging stems more frequently used after SHS fixation. Stem type did not significantly affect survivorship, complications, or clinical outcomes, with survivorship free from stem-related failure of 100% and free from all-cause failure of 89% at a mean follow-up of 69 months. Patients who received cemented stems were older and more likely to require transfusion and postoperative use of an assistive device, reflecting medical and functional differences rather than implant performance.

This study is novel in several ways. First, prior studies evaluated cTHAs relative to primary THA while our is the first to our knowledge to compare cemented, uncemented metaphyseal-engaging and uncemented diaphyseal-engaging stems after cTHA. in addition, our study evaluates the function of these stems from clinical and radiographic viewpoint in a manner not previously employed by prior studies of cTHA outcomes. Moreover, our study is the first to our knowledge to propose a preliminary, data-driven treatment algorithm that may assist surgeons in determining stem choice at the time of cTHA and serve as a foundation for future validation studies. While the algorithm demonstrated high predictive accuracy within our limited sample (98%, 50 of 51 cases), it should be considered exploratory and hypothesis-generating.

We aimed to determine the association between femoral stem fixation used in cTHA and previous proximal femoral fracture fixation construct and pattern as we hypothesized that there would be an association between these variables. It was noted that diaphyseal-engaging uncemented stems were the stem of choice for patients who had previous CMN (68%), while metaphyseal-engaging uncemented stems were predominantly used in patients who had previous SHS (57%). Cemented stems were used in relatively fewer patients than uncemented stems, although when they were used, they were generally implanted in patients who had previous CMN. This analysis was not thoroughly performed in previous studies although some studies reported which stems were used in certain subgroups. Bercik et al. reported the use of diaphyseal-engaging stems in 100% of cTHAs after CMN and 75% of cTHAs after SHS [32]., while Marcantonio et al. described use of predominantly diaphyseal-engaging stems during cTHA after CMN fixation in 15 hips and predominantly metaphyseal-engaging stems after SHS fixation in 17 hips [21]. In the study by Yuan et al., modular diaphyseal-engaging stems appeared to be used more frequently following CMN (22%) than extramedullary fixation (4%), although fully coated (monobloc diaphyseal-engaging) stems were used in 5% of hips after CMN and 15% of hips after extramedullary fixation [7]. Standard metaphyseal-engaging stems were used in 10% of cTHAs after extramedullary fixation and were not used after CMN fixation. Cemented stems were used more frequently in the current study relative to other studies.

In the analysis of the association between femoral stem fixation used during cTHA and previous fracture pattern in our study, diaphyseal-engaging uncemented stems were the predominant stem type used in patients with previous intertrochanteric, subtrochanteric, or multiple/complex fractures, while metaphyseal-engaging uncemented stems were the predominant stem type used in patients with previous femoral neck fractures. Determination of stems used in patients based on fracture pattern has not been routinely reported in other studies to date. It should be noted that fracture fixation and fracture morphology naturally tend to correlate.

With respect to survivorship after cTHA, we found that survivorship free from stem-related failure requiring stem revision was 100%, and survivorship free from all-cause failure requiring reoperation was 89% at mean follow-up of 5.8 years. Reoperations were performed for infection and instability. The implant survivorship found in this study is relatively consistent with previously published literature that demonstrated that survivorship following cTHA is lower than after standard primary THA,^8^ and similar to revision THA [9–14]. In previous studies of cTHA, relatively high rates of revision surgery and complications were demonstrated. In a large study of 56,522 patients, Smith et al. reported a rate of revision surgery that was higher in cTHA compared to primary (non-conversion) THA (8% versus 4%).^8^ They found significantly higher rates of infection, dislocation, revision for infection, and revision for dislocation following cTHA than primary THA. Yuan et al. reported 5-year survivorship free from revision of 94% and 95% for cTHA after previous fixation using a CMN in 41 cases and an extramedullary device in 70 cases, respectively [7]. Revisions were performed for infection, instability, and aseptic loosening of the femoral component. In a multicenter study, Pui et al. found that orthopaedic complications – defined as fracture, aseptic loosening, dislocation, abductor deficiency, heterotopic ossification, or nerve injury – occurred in 16% of cTHAs, and periprosthetic joint infection occurred in 2% of cTHAs [15]. These authors noted a significantly higher rate of orthopaedic complications in those with previous CMN compared to SHS (29% versus 8%). Godoy-Monzon reported complications in 14% of 28 patients treated with cTHA using a modular stem [33]. These included two dislocations, one superficial wound infection and one abductor deficiency.

In our evaluation of the impact of femoral stem type on survivorship, clinical outcomes, and PROMs following cTHA, there was no effect of stem type found in the analysis of stem-related failures. However, given the small subgroup sizes (32 diaphyseal-engaging, 10 metaphyseal-engaging, and 9 cemented stems), particularly for PROMs where data were available for only 20 patients, the precision and generalizability of these statistical comparisons are limited. We did find that patients with cemented stems were more likely to require blood transfusion and use an assistive device postoperatively. This is likely due to the fact that the patients with cemented stems were older and therefore likely less healthy and more decompensated. Furthermore, instability and Vancouver C periprosthetic fractures may represent complications partially attributable to stem design or fixation, and inclusion of such cases as stem-related failures could potentially influence the observed associations.

Otherwise, femoral stem type had no significant impact of on survivorship or any of the outcomes evaluated in our current study. This finding differs from the work of Shi et al.^20^ who found that patients with diaphyseal-engaging stems were more likely to have complications, intraoperative femoral fractures, increased operative time, and increased blood loss. Shi et al. did not have a group of patients with cemented femoral stems in their study. The discrepancy between studies may relate to differences in surgical technique and the frequency of diaphyseal-engaging stem use across institutions, as greater familiarity with this fixation method may reduce the risk of intraoperative complications reported elsewhere.

Although it could potentially be concluded that the type of stem fixation does not affect outcome in cTHA, it is quite possible that there were no differences noted between groups given that the appropriate stem types were used in patients with specific characteristics. In primary THA, factors such as femoral morphology and canal filling have been shown to influence stem stability and long-term fixation, and similar biomechanical principles likely apply to cTHA, where canal geometry, bone quality, and degree of prior hardware violation may affect stem engagement and survivorship [34]. 

The authors of this study believe that performing a detailed preoperative evaluation of different patient’s characteristics may be associated with improved survivorship. This could be divided into patients medical variables including age, sex, chronic medication use, smoking status and even alcohol consumption which are all associated with bone quality. Comorbidities that contribute to gait instability may influence the choice of stem fixation method. History of patients’ bone density, quality and femoral canal morphology as well as bony defects and stress risers associated with previous fixation hardware may influence stem length and fixation type. These variables could impact survivorship, and these should be considered when choosing a stem for cTHA.

The factors that should dictate the type of stem fixation used to optimize survivorship and outcome following cTHA have not been elucidated to date in the literature. Proximal femoral fracture pattern and the type of fracture fixation construct used are likely to be important factors which each of these likely serving as a proxy for each other. Using our current study, we put forth a novel treatment algorithm for the selection of femoral stem in cTHA. Our study and the work of others suggests that there is a trend for surgeons to use diaphyseal-engaging stems in patients who were previously treated with CMN fixation, while metaphyseal-engaging stems are predominantly used in those with femoral neck fractures [21]. It is anticipated that one reason for this is that CMNs are commonly used for treating intertrochanteric fractures, subtrochanteric fractures, and complex/multiple fractures and these fractures more significantly alter the area of bone in the metaphysis than femoral neck fractures, thus warranting use of diaphyseal engaging stems which do not rely on metaphyseal fixation which might be compromised. On the other hand, SHS (and other extramedullary devices and/or screws) are commonly used for treating femoral neck fractures, including basicervical fractures, and these fractures generally do not alter the metaphyseal anatomy as significantly. Therefore, adequate engagement can be generated using a metaphyseal-engaging stem. Cephalomedullary devices such as CMNs generate more alteration in the metaphyseal bone due to canal reaming as well as both the nail and screw fill space within the metaphysis may lead to development of sclerotic margins at the interface between bone and implant. SHSs do not violate the metaphysis as much as only part of the implant is present within the femur while the side plate is extramedullary. Proximal femoral geometry is another factor to consider. Geometry can be altered at the time of fracture, during reduction, and with fracture healing. When the metaphysis is significantly disrupted, it is anticipated that a metaphyseal-engaging stem may not be as ideal of a construct as a diaphyseal-engaging stem. In our cohort, the observed relationship between femoral stem type and fracture pattern likely reflects preoperative surgical planning rather than a true independent association, as stem choice was inherently influenced by the fracture morphology and prior fixation construct. Malunion of an intertrochanteric, subtrochanteric, or complex fracture pattern would potentially decrease the volume of bone available for engagement and fixation. Sclerotic bone in the area would be less likely to allow for stem engagement and fixation. The geometry of the femoral diaphysis should also be considered. In those patients with very large diameter canals, it is possible that a diaphyseal-engaging stem might not adequately engage the canal isthmus and there could be consideration for use of a cemented stem. Further study is required in this field to further elucidate and evaluate factors that should drive stem selection in cTHA. Moreover, future studies should consider testing our exploratory algorithm.

This study has some limitations. First, given that this was a retrospective study, only associations between variables could be determined and relationships analyzed between factors and outcomes could not be interpreted as causal. Second, we only determined the relationship between femoral stem used in cTHA and previous proximal femoral fracture fixation construct, and separately between femoral stem used in cTHA and previous proximal femoral fracture pattern. However, we did not discern the interplay between all three variables. Third, it is possible that our study is underpowered in regard to elucidating differences in outcomes in our stem type groups. Further research with multicenter or large database studies could be beneficial.

## Conclusions

In this retrospective study of cTHA patients, stem fixation type was associated with previous fracture pattern and fixation construct. Survivorship free from stem-related failure requiring stem revision was 100% and survivorship free from all-cause failure was 89%. Patients who received cemented stems were older and more likely to require transfusion and postoperative use of an assistive device. There were no other differences in postoperative complications, clinical outcomes or PROMs between stem groups. We propose a novel treatment algorithm for femoral stem selection in conversion THA. This algorithm is preliminary and requires validation in future studies with longer-term follow-up and larger patient samples to further elucidate the factors that impact femoral stem choice and outcomes in cTHA.

## Supplementary Information

Below is the link to the electronic supplementary material.

Supplementary Material 1

## Data Availability

The individual patient data that support the findings of this study are not openly available due to reasons of sensitivity. Data are located in controlled access data storage at Hospital for Special Surgery.
