# Supplementary material for: Does femoral stem choice associate with survivorship and clinical outcomes after conversion total hip arthroplasty? A retrospective analysis and novel treatment algorithm
Source: Arch Orthop Trauma Surg. 2026 Feb 24;146(1):76. doi: 10.1007/s00402-025-06181-4 (PMC12932333; doi:10.1007/s00402-025-06181-4)
Supplement: Supplementary file 1 — Supplementary Material 1 [file 402_2025_6181_MOESM1_ESM.docx]

**SUPPLEMENTAL TABLES AND FIGURES**

**Supplemental Table 1. Primary Diagnosis based on Femoral Stem Implanted in Conversion Total Hip Arthroplasty.**

|  | **All Stems**  **N (%)^1^** | Cemented  N (%)^2^ | Uncemented N (%)^2^ | Metaphyseal N (%)^2^ | Diaphyseal  N (%)^2^ | Modular  N (%)^2^ | Monoblock  N (%)^2^ |
| --- | --- | --- | --- | --- | --- | --- | --- |
| Arthritis | **28 (54.9%)** | 3 (10.7%) | 25 (89.3%) | 8 (28.6%) | 17 (60.7%) | 8 (47.1%) | 9 (13.6%) |
| Avascular necrosis | **8 (15.7%)** | 4 (50.0%) | 4 (50.0%) | 2 (25.0%) | 2 (25.0%) | 2 (25.0%) | 0 (0.00%) |
| Nonunion without screw cut-out | **8 (15.7%)** | 1 (12.5%) | 7 (87.5%) | 0 (0.00%) | 7 (87.5%) | 2 (25.0) | 5 (62.5%) |
| Nonunion with screw cut-out | **5 (9.8%)** | 1 (20.0%) | 4 (80.0%) | 0 (0.00%) | 4 (80.0%) | 4 (80.0%) | 0 (0.00%) |
| Malunion | **2 (3.9%)** | 0 (0.00%) | 2 (100.0%) | 0 (0.00%) | 2 (100.0%) | 1 (50.0%) | 1 (50.0%) |
| All diagnoses | **51** | 9 (17.6%) | 42 (82.4%) | 10 (19.6%) | 32 (62.7%) | 17 (33.3%) | 15 (29.4%) |

^1^Number and percentage of patients treated for each diagnosis

^2^Number and percentage of stems used for each indication.

**Supplemental Table 2. Tonnis Grading of Hip Osteoarthritis.**

| **Tonnis Grade** | **Number of patients (%)** |
| --- | --- |
| 0 | 5 (9.8%) |
| 1 | 7 (13.7%) |
| 2 | 15 (29.4%) |
| 3 | 24 (47.1%) |

**Supplemental Table 3. All-Cause Failures based on Femoral Stem Type Implanted in Conversion THA.**

| **Failure Mode** | **Type of femoral stem** | **Stem Fixation** | **Treatment** |
| --- | --- | --- | --- |
| Infection | Double-taper/fit and fill | Metaphyseal-engaging uncemented | Head+liner revision/debridement |
| Infection | Modular | Diaphyseal-engaging uncemented | Debridement |
| Infection | Monobloc | Diaphyseal-engaging uncemented | Head+liner revision/debridement |
| Instability | Modular | Diaphyseal-engaging uncemented | Head+liner revision (dual mobility) |
| Instability | Cemented | Cemented | Head+liner revision (dual mobility)  Acetabular revision |

Abbreviations: THA, total hip arthroplasty

**Supplemental Table 4. Survivorship based on Femoral Stem Implanted in Conversion Total Hip Arthroplasty.**

| **Femoral stem type used in conversion THA** | **n** | **Survivorship free from stem-related revision** | **Survivorship free from all-cause failure (95% CI)** | **P-value** |
| --- | --- | --- | --- | --- |
| *All* | 51 | 100% | 89% (79-98%) | 0.8 |
| Uncemented | 42 | 100% | 95% (89-100%) |  |
| Cemented | 9 | 100% | 89% (68-100%) |  |
| *Uncemented* | 42 | 100% | 95% (89-100%) | 1.0 |
| Diaphyseal-engaging | 32 | 100% | 94% (85-100%) |  |
| Metaphyseal-engaging | 10 | 100% | 86% (60-100%) |  |
| *Diaphyseal-engaging* | 32 | 100% | 94% (85-100%) | 0.70 |
| Modular | 17 | 100% | 94% (93-100%) |  |
| Monobloc | 15 | 100% | 93% (81-100%) |  |

*P value calculated using Log-Rank test to compare groups.*

*Significant P values in bold.*

Abbreviations: THA, total hip arthroplasty; CI, confidence interval

**Supplemental Table 5. Subsidence of Femoral Stems Used in Conversion Total Hip Arthroplasty.**

| **Femoral stem type used in conversion THA** | **Mean subsidence**  **(mm)** | ***P* value** | **Subsidence ≥5 mm**  **(n [%])** | ***P* value** |
| --- | --- | --- | --- | --- |
| All | 1.2 ± 1.8 | 0.12 | 4 (8.0%) | 0.33 |
| Uncemented | 1.3 ± 2.1 |  | 4 (9.8%) |  |
| Cemented | 0.4 ± 0.9 |  | 0 (0.0%) |  |
| Uncemented | 1.2 ± 1.8 | 0.30 | 4 (9.8%) | 0.23 |
| Diaphyseal-engaging | 1.6 ± 2.2 |  | 4 (12.9%) |  |
| Metaphyseal-engaging | 0.6 ± 0.7 |  | 0 (0.0%) |  |
| Diaphyseal-engaging | 1.6 ± 2.2 | 0.14 | 4 (12.9%) | 0.35 |
| Modular | 1.6 ± 1.5 |  | 3 (18.8%) |  |
| Monobloc | 1.5 ± 2.9 |  | 1 (6.7%) |  |

*P values calculated using independent sample T-test, Mann-Whitney U, or Fisher’s exact tests to compare groups.*

*Significant P values in bold.*

Abbreviations: THA, total hip arthroplasty


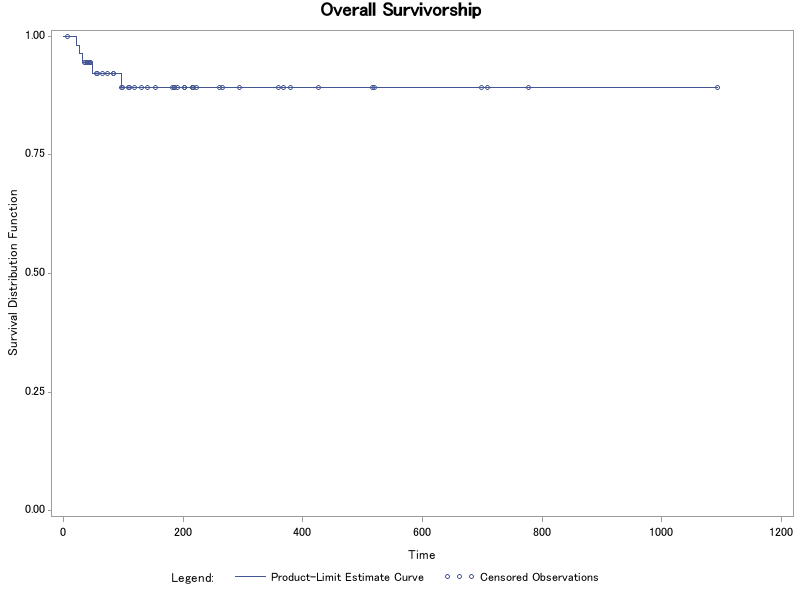


**Supplemental Figure 1.** Survivorship free from all-cause failure of conversion total hip arthroplasty.


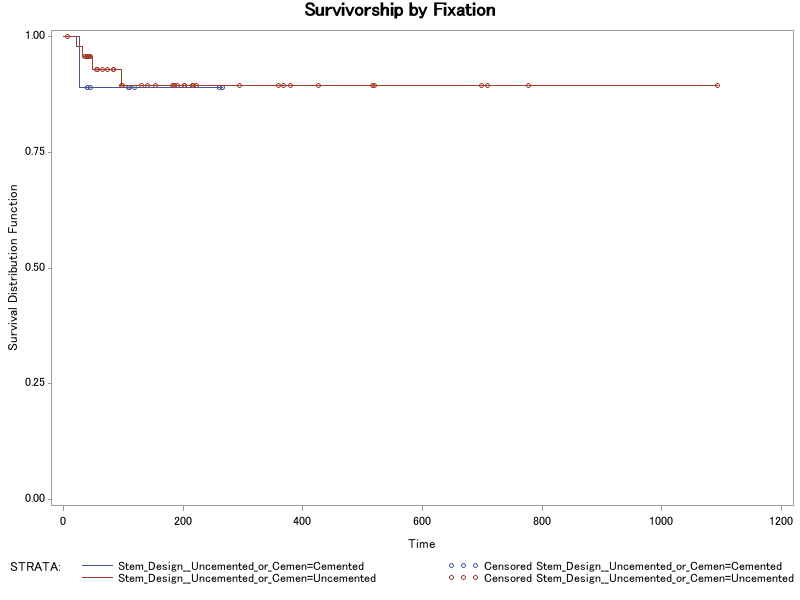


**Supplemental Figure 2.** Survivorship free from all-cause failure of conversion total hip arthroplasty with uncemented and cemented femoral stems.

Blue line represents patients with cemented femoral stems used at the time of conversion total hip arthroplasty.

Red line patients with uncemented femoral stems used at the time of conversion total hip arthroplasty.


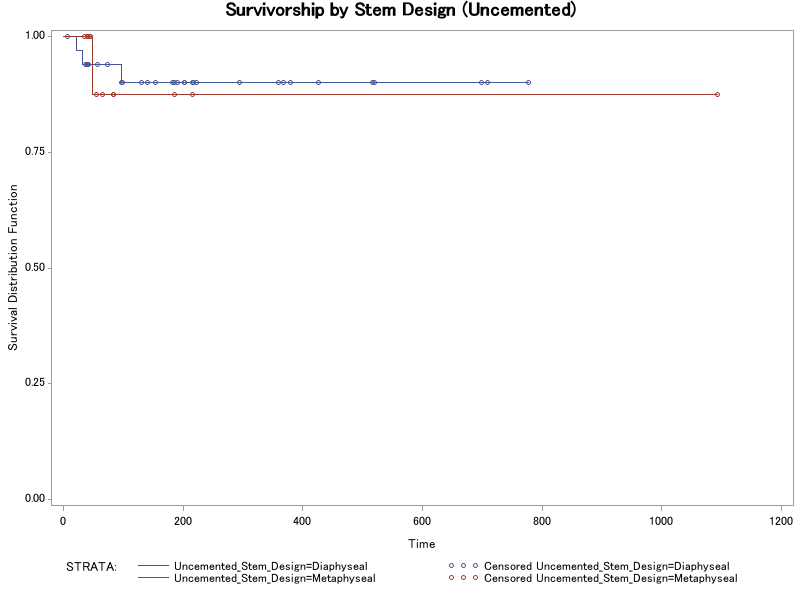


**Supplemental Figure 3.** Survivorship free from all-cause failure of conversion total hip arthroplasty with diaphyseal-engaging uncemented stems and metaphyseal-engaging stems.

Blue line represents patients with uncemented diaphyseal-engaging femoral stems used at the time of conversion total hip arthroplasty.

Red line represents patients with uncemented metaphyseal-engaging femoral stems used at the time of conversion total hip arthroplasty.


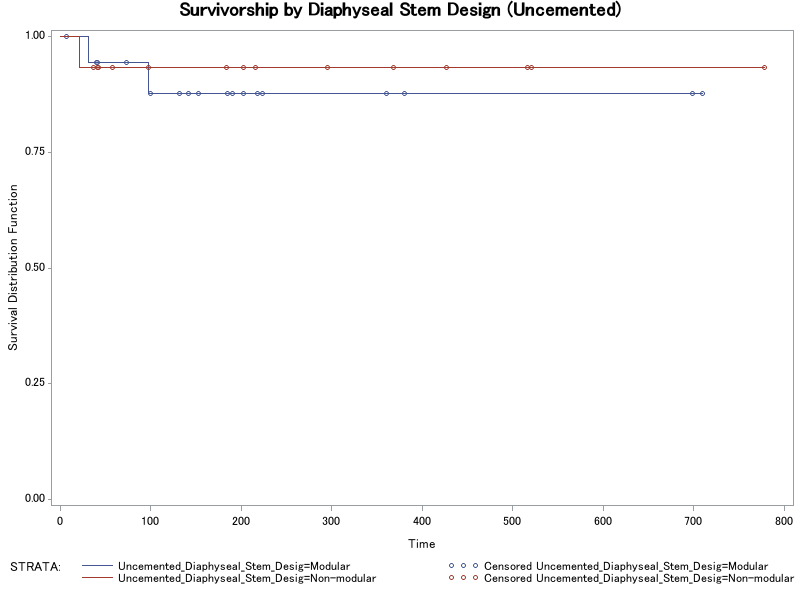


**Supplemental Figure 4.** Survivorship free from all-cause failure of conversion total hip arthroplasty with modular diaphyseal-engaging uncemented stems and monobloc diaphyseal-engaging uncemented stems.

Blue line represents patients with modular uncemented diaphyseal-engaging femoral stems used at the time of conversion total hip arthroplasty.

Red line represents patients with monobloc uncemented diaphyseal-engaging femoral stems used at the time of conversion total hip arthroplasty.
